# Subepicardial adipose genes contribute to the deterioration of heart failure preserved ejection fraction

**DOI:** 10.3389/fcvm.2025.1501397

**Published:** 2025-02-21

**Authors:** Ruiying Zhang, Man Wang, Yuheng Lang, Jiaqi Zhang, Yuchao Wang, Han Zheng, Yue Zheng, Bingyang Zhou

**Affiliations:** ^1^ Department of Emergency and Critical Care, Tianjin University Chest Hospital, Tianjin, China; ^2^ Department of Emergency and Critical Care, Tianjin Chest Hospital, Tianjin, China; ^3^ Tianjin Key Laboratory of Cardiovascular Emergency and Critical Care, Tianjin Municipal Science and Technology Bureau, Tianjin, China; ^4^ Department of Cardiology, Tianjin University Chest Hospital, Tianjin, China; ^5^ Department of Cardiology, Tianjin Chest Hospital, Tianjin, China; ^6^ Department of Heart Center, The Third Central Hospital of Tianjin, Tianjin, China; ^7^ Department of Heart Center, Tianjin Key Laboratory of Extracorporeal Life Support for Critical Diseases, Tianjin, China; ^8^ School of Medicine, Nankai University, Tianjin, China; ^9^ State Key Laboratory of Membrane Biology and Tsinghua-Peking Center for Life Sciences, Beijing Advanced Innovation Center for Structural Biology, School of Life Sciences, Tsinghua University, Beijing, China

**Keywords:** heart failure, visceral adipocyte genes, HFpEF, SASPs, macrophage, human sub-epicardial tissues

## Abstract

**Background:**

The mortality of patients with acute myocardial infarction (MI) raised rapidly in last decade and obesity are becoming the major cause to CAD progression, thus inducing heart failure preserved ejection fraction (HFpEF). However, why visceral adipocytes show different effects on healthy and ageing cardiomyocytes is less known.

**Methods:**

GSE251971 was downloaded and Venn diagram between visceral adipocyte genes genes and DEGs was performed to obtain visceral adipocyte-associated DEGs in heart failure. Protein-protein interaction (PPI) network was constructed to obtain the hub genes utilizing the Cytoscape plugin Cytohubba. The hub genes and their interactions were analyzed using NetworkAnalyst 3.0 and for validation, the hub genes expressions were analyzed using Single-cell sequencing data, cell lines and human sub-epicardial tissues and blood samples.

**Results:**

Using Venn diagram, 71 visceral adipocyte-associated DEGs were identified. Nine hub genes were obtained, including OGN, SELL, FOS, NKG7, LOX, HBB, CXCL9, CP and ALOX5. Single-cell sequencing demonstrated all hub genes were highly expressed in human hypertrophic cardiomyopathy and ischemic cardiomyopathy patients with end-stage heart failure. The related OGN, FOS, NKG7 and ALOX5 mRNA expressions were significantly highly expressed in sub-epicardial tissues in HFpEF patients. AUCs of OGN, FOS and ALOX5 were 0.902, 0.795 and 0.730, and the AUC of joint ROC of OGN, FOS and ALOX5 was 0.946. Additionally, FOS, ALOX5 and OGN expressions were increased at follow up 1 year recurrence, while decreased at follow up 2 year recurrence. Mechanically, FOS and ALOX5 were highly expressed in macrophages under hypoxia, while OGN was highly expressed in fibroblasts under hypoxia. SASPs, including IL1α, IL1β, IL6 and TNFα, decreased in hypoxic macrophages after FOS and ALOX5 knockdown or both. Also, SASPs decreased in hypoxic fibroblasts after OGN knockdown. These results suggested that FOS, ALOX5 and OGN may affect cell senescence after hypoxia, thus inducing myocardial infarction and HFpEF progression.

**Conclusion:**

The screened hub genes, including OGN, FOS and ALOX5, were validated using single-cell sequencing data, cell lines and human samples, which can be therapeutic targets for the treatment to cell senescence under hypoxia and prediction to heart failure progression to HFpEF.

## 1 Introduction

Coronary artery disease (CAD), especially ischemic cardiomyopathy-induced heart failure, still results in the leading death in low- and middle-income countries (1). The mortality of patients with acute myocardial infarction (MI) raised rapidly in last decade and obesity are becoming the major cause to CAD progression, thus inducing heart failure preserved ejection fraction (HFpEF) (2, 3). Conservatively estimated, nowadays 330 million people develop heart diseases in China and young patients with bad habits have higher long-term cardiovascular and all-cause mortality, who need more aggressive secondary prevention (2, 3).

The effects of visceral adipocytes were demonstrated to show more positive effects on cardiomyocytes after MI than we ever thought. For instance, pericardial adipose tissue regulated granulopoiesis and increased cardiac functions after MI (4). IL6/adiponectin/HMGB1 feedback loop mediated subepicardial adipocyte and macrophage crosstalk and M2 polarization after MI (5). Inhibiting macrophage receptor CCR2 strengthened the adiponectin effects against myocardial injury after infarction (6). In addition, re-activation of the visceral adipocytes in epicardium resulted in cardiac remodeling after myocardial injury via paracrine secretion (7).

Ageing is a phenotype characterized by complex physiological, cellular and molecular changes (8). Adipose tissues are as a linchpin of organismal ageing (9) and showed negative effects on ageing cardiomyocytes under stress (10). Visceral adipocyte ageing in heart drove metabolic decline and induced cardiomyocytes and macrophages ageing under stress (9, 10), which was different from the effects on healthy cardiomyocytes. Cellular senescence demonstrates a stable cell cycle arrest correlated to typical morphological cellular changes and senescence-associated secretory phenotype (SASP) (11). Nowadays, why visceral adipocytes show different effects on healthy and ageing cardiomyocytes is less known. The visceral adipocyte-associated genomic signature reflecting epicardial adipose and immune infiltration in heart failure is also less known.

In this study, GSE251971 was downloaded and visceral adipocyte-associated differential expressed genes (DEGs) in heart failure were obtained. The protein-protein interaction (PPI) network of DEGs was performed to explore the hub genes. Single-cell sequencing data and human sub-epicardial adipose tissue samples were used to validate the expression of the hub genes in heart failure. The collected human blood samples and 3T3 and RAW 264.7 cell lines were used to analyze the effects of hub genes on heart failure deterioration, which may be potential targets of SASPs and prevent MI progression and heart failure deterioration.

## 2 Methods

### 2.1 Data source and data processing

Utilizing the keywords “heart failure” in “homo sapiens”, GSE251971 was found. There were 6 myocardial biopsies of patients with rhythm disturbance and chronic heart failure induced by ischemic aetiology before treating with the implantation of support device Optimizer (OPT) and 6 myocardial biopsies after undergoing the implantation of OPT (12). The DEGs were analyzed using the GEO2R. A log2|FC| > 1 and an adjusted *P* value < 0.05 were considered significant.

The visceral adipocyte genes were downloaded from the Genecard database (https://www.genecards.org/). Venn diagram between visceral adipocyte genes genes and DEGs was performed to obtain visceral adipocyte-associated DEGs.

### 2.2 Enrichment analyses

Gene Ontology and Kyoto Encyclopedia of Genes and Genomes analysis were carried out using R package clusterProfiler (13–15). Statistical significance was set at *P* < 0.05 was considered significant.

### 2.3 PPI network analysis and hub genes

To explore the hub genes of visceral adipocyte-associated DEGs, STRING database (https://string-db.org) was used with a combined score >0.4 (16). The nodes were analyzed using Cytoscape v.3.7.1 (17). PPI network analysis was constructed to obtain the hub genes utilizing the Cytoscape plugin Cytohubba and Top10 MCC hub genes and top10 Degree hub genes were obtained.

### 2.4 The hub visceral adipocyte-associated DEGs and their interactions

NetworkAnalyst 3.0 is a comprehensive network visual analyzed platform for gene expression analysis (18). The hub visceral adipocyte-associated DEGs and their interactions were analyzed using NetworkAnalyst 3.0. Specifically, transcription factors (TFs)-hub visceral adipocyte-associated DEGs interactions were shown using the TRRUST database (a reference database of human transcriptional regulatory interactions). Drugs-hub visceral adipocyte-associated DEGs interactions were shown using the DrugBank database (Version 5.0). Left ventricle tissue-specific PPI were shown using the DifferentialNet database (Filter is 15), which shows the differential protein-protein interactions across human tissues. The top10 Degree hub genes of left ventricle tissue-specific PPI were obtained utilizing the Cytoscape plugin Cytohubba Degree Method.

### 2.5 Single-cell sequencing data validation

Single-cell sequencing data were obtained from the Single Cell Portal (https://singlecell.broadinstitute.org/) to explore the hub gene expression in different cell lines (SCP1303 and SCP1849). SCP1303 and SCP1849 were the single-cell sequencing data about human hypertrophic cardiomyopathy and ischemic cardiomyopathy patients with end-stage heart failure, respectively.

### 2.6 Human sample validation

The human sub-epicardial adipose tissue samples were collected during CABG surgery to validate the diagnostic value of the hub genes. All the myocardial tissue samples were collected from the proximity of the culprit vessel during the CABG surgery. The human blood samples were collected at 1d after admission.

The cohorts with CAD were divided into two groups according to left ventricular ejection fraction by ultrasonic detection: HFpEF group and other patients with mild-reduced or reduced heart failure named OHF. The baseline characteristics, such as gender, age and prior medical history, were also collected. The inclusion criteria were as follows: (1) the patients under CABG surgery; (2) the sub-epicardial adipose tissue samples can be collected; and (3) the patients' information and the laboratory examinations were completed. The exclusion criteria were as follows: (1) the history of nephropathy, hepatopathy, diabetic oculopathy, brain damage and tumor; (2) cardiac arrest or patients with ECPR; (3) Aortic insufficiency or aortic dissection; and (4) Uncontrollable bleeding. The first admission HFpEF patients were followed up 2 years and the recurrence was recorded. The protocol and patient consents were approved by Tianjin Third Central Hospital.

### 2.7 Cell source and processing

3T3 (fibroblast) and RAW264.7 (macrophage) cell lines were purchased from Chinese Academy of Medical Sciences and was cultured in DMEM medium containing 10% (v/v) fetal bovine serum (FBS) in a 37℃, 5% CO2 incubator before experiment.

The OGN expression in 3T3 cell was knockdown by using 9 ul lipo2000 (11668-019, Invitrogen), adding each 50 nM siRNA 3 ul (mmu-OGN-1, mmu-OGN-2 and mmu-OGN-3, RIBIBIO, China) and 250 ul OptiMEM medium (31985-070, Gibco) per well. The nonsense sequences were used as the negative control (210011, Ubigene). FOS and ALOX5 knockdown RAW 264.7 macrophages were obtained using adenovirus (m-FOS-shRNA-GFP-Puro and m-ALOX5-shRNA-GFP-Puro from 293 T cells, 6.57 × 108 TU/ml, Genechem, Shanghai). Cells were cultured in six-well plates (5 × 105 cells per well), transfected using shRNA (MOI = 10), and screened using puromycin (2 ug/ml, Genechem, Shanghai) to obtain the infected RAW 264.7 macrophages.

3T3 and RAW 264.7 cells were maintained in high glucose Dulbecco's modified Eagle's medium (Gibco, USA) containing 10% fetal bovine serum (AusgeneX, Australia), 1% penicillin/streptomycin (Solarbio, China) at 37°C in an atmosphere of normal condition (5% CO2% and 95% air) or hypoxia condition (5% CO2, 94% N2, and 1% O2).

### 2.8 qPCR analysis

The hub genes were validated using a human sub-epicardial adipose tissue. qRT-PCR was performed using TRIzol and TB Green method (TaKaRa, RR820). β-actin was the reference gene and the 2-ΔΔCt method was utilized. FOS, ALOX5 and OGN mRNA expressions were analyzed in macrophage and fibroblast under hypoxia using qRT-PCR. The knockdown efficiency was also measured using qRT-PCR. The primer details were shown in Supplementary Table 1.

### 2.8 ELISA

Human FOS ELISA Kit (ab264626, Abcam), Human ALOX5 ELISA Kit (LS-F35407, LsBio) and Human OGN ELISA Kit (CSB-EL016314HU, CusaBio) were used to determine the expressions in the human blood samples of HFpEF patients and their recurrence.

Mouse IL-1α ELISA Kit (EK201A, MULTI SCIENCES), mouse IL-1β ELISA Kit (EK201BHS, MULTI SCIENCES), mouse IL-6 ELISA Kit (PI326, Beyotime) and mouse TNFα ELISA Kit (PT512, Beyotime) were used to determine the expressions in the cell culture supernatants to analyze the SASPs and cell senescence.

### 2.9 Statistical analysis

All data were shown as mean ± SEM after normal distribution analysis. The Shapiro–Wilk normality test, Welch's *t*-test (two groups), One-way ANOVA, and Two-way ANOVA were used for statistical analysis. ROC analysis was performed and the area under curves (AUCs) were obtained in human blood samples to investigate the diagnostic value between HFpEF patients and OHF patients. A multiple comparison *P* < 0.05 was considered significant.

## 3 Results

### 3.1 Visceral adipocyte-associated DEGs and functional enrichment analysis

Using GEO2R, 304 DEGs were identified in the GSE251971 dataset (Supplementary Figure 1). The DEGs were mainly involved in oxygen transport, hydrogen peroxide catabolic process, NADPH oxidase complex, peroxidase activity, etc. (Supplementary Figure 2; Supplementary Table 2).

**Figure 1:**
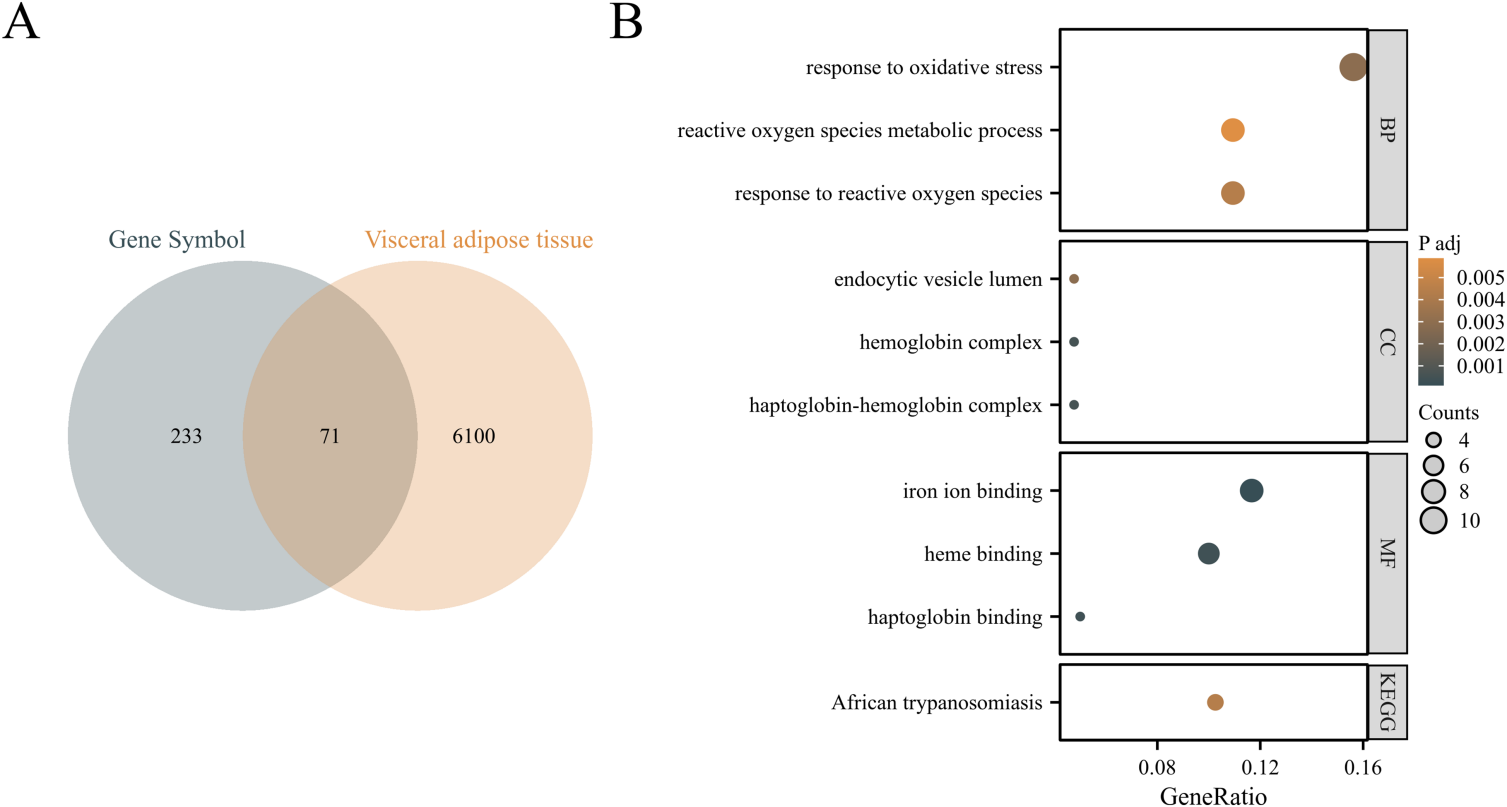
Identification of visceral adipocyte-associated DEGs in chronic heart failure patients. **(A)** Venn diagram showed DEGs between heart failure-related DEGs and visceral adipocyte genes. **(B)** The GO/KEGG pathways enriched by DEGs in chronic heart failure patients. BP, biological process; CC, cellular component; MF, molecular function; KEGG, Kyoto encyclopedia of genes and genomes.

Using Venn diagram, 71 visceral adipocyte-associated DEGs were identified (Figure 1A). They were mainly enriched in response to reactive oxygen species, reactive oxygen species metabolic process, endocytic vesicle lumen, oxygen binding, etc. (Figure 1B; Supplementary Table 3).

### 3.2 Protein-protein interaction (PPI) network analysis

**Figure 2:**
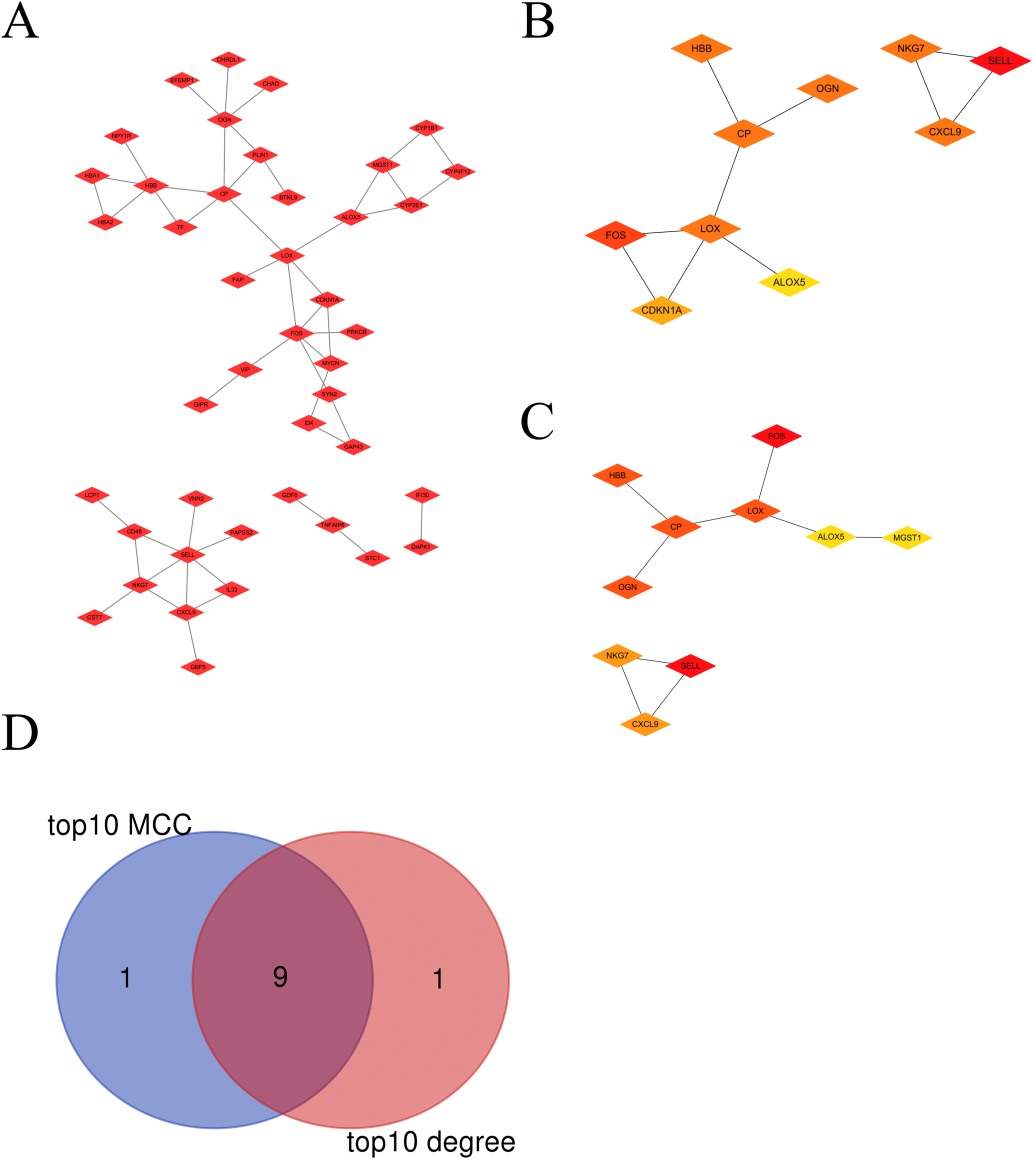
The hub visceral adipocyte-associated DEGs were obtained. **(A)** The protein-protein interaction network of visceral adipocyte-associated DEGs were showed. **(B,C)** The hub DEGs were obtained using Cytoscape Cytohubba plugin MCC method **(B)** and degree method. **(C,D)** The Venn diagram between MCC top10 genes and degree top10 genes.

A PPI network was constructed (Figure 2A). Using the Cytoscape plug-ins MCC and Degree, the top 10 genes were identified (Figures 2B,C). A Venn diagram was utilized to obtain the intersection among the top 10 MCC and top 10 Degree genes. Nine hub genes were the same, including OGN, SELL, FOS, NKG7, LOX, HBB, CXCL9, CP and ALOX5 (Figure 2D).

**Figure 3:**
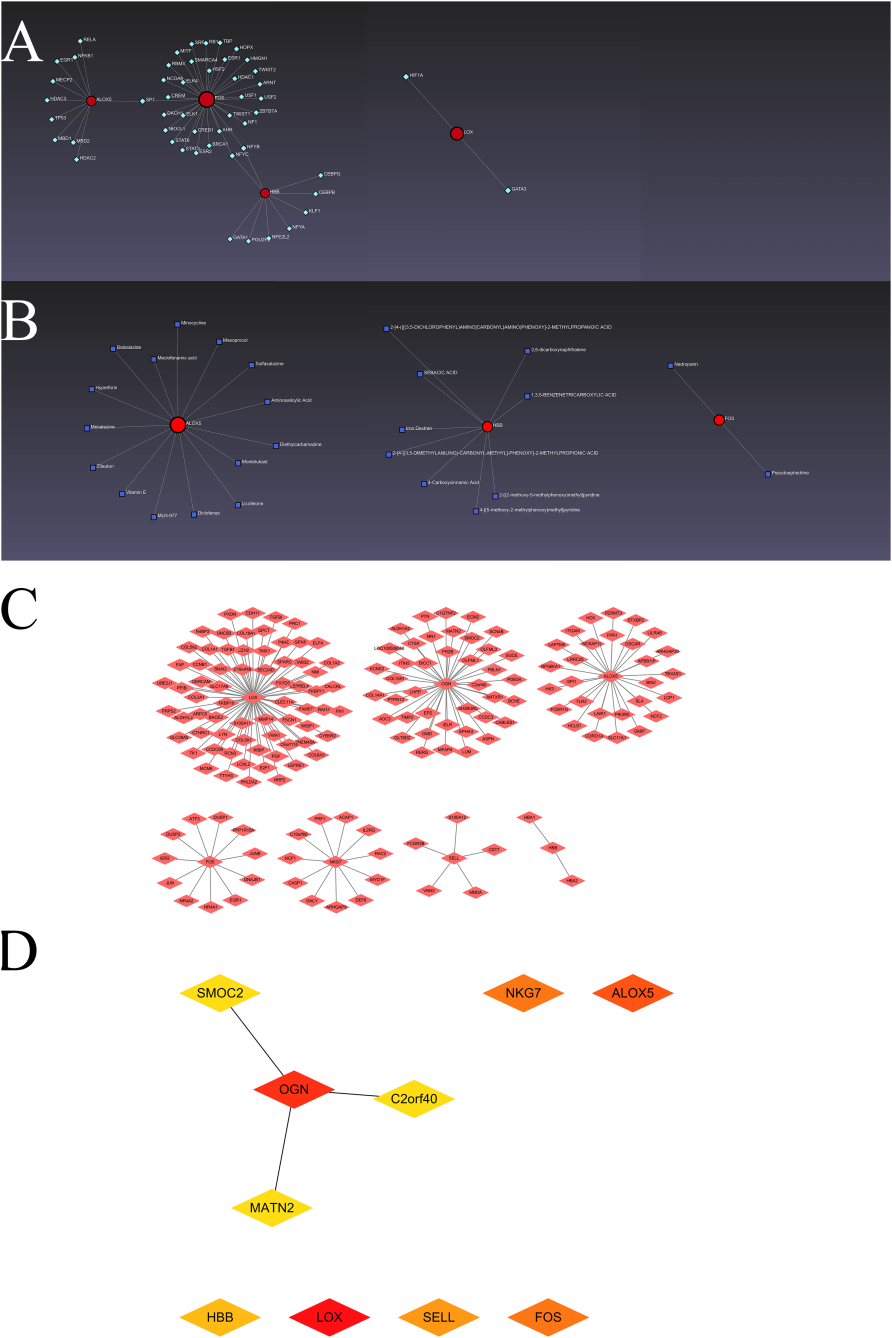
The hub visceral adipocyte-associated DEGs and the interactions. **(A)** The networks of transcription factors-hub visceral adipocyte-associated DEGs interactions. **(B)** The networks of drugs-hub visceral adipocyte-associated DEGs interactions. **(C)** The protein-protein interaction network of left ventricular tissue-specific co-expression. **(D)** The left ventricular tissue-specific co-expression hub DEGs were obtained using Cytoscape Cytohubba plugin degree method.

The interactions of transcription factors and hub visceral adipocyte-associated DEGs, including ALOX5, FOS, HBB and LOX, were shown using the TRRUST database (Figure 3A). The interactions of drugs and hub visceral adipocyte-associated DEGs, including ALOX5, FOS and HBB, were shown using the DrugBank database (Version 5.0) (Figure 3B). Left ventricle tissue-specific PPI were shown and the top10 Degree hub genes of left ventricle tissue-specific PPI were obtained (Figures 3C,D).

### 3.3 Single-cell sequencing analysis

**Figure 4:**
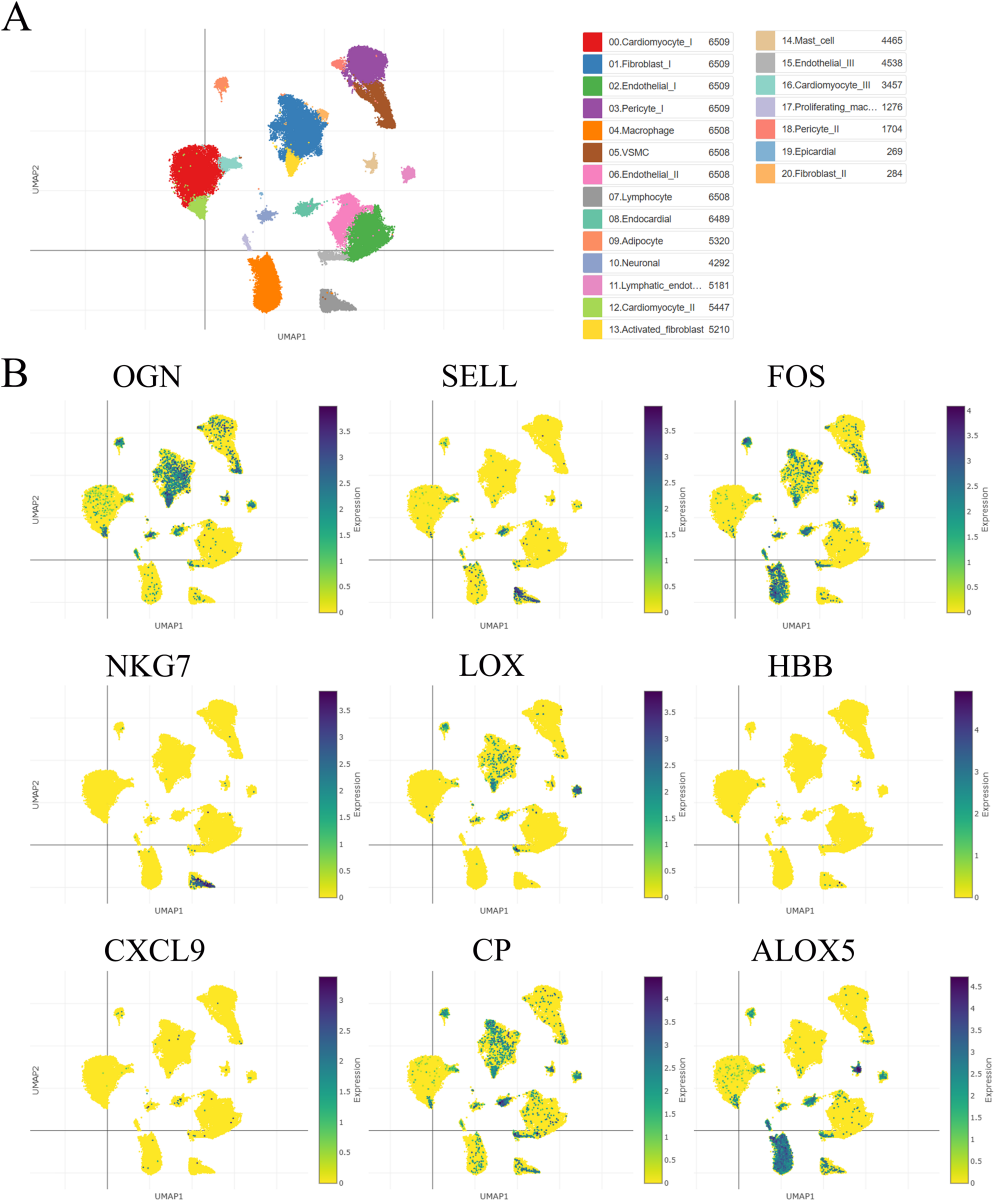
The hub visceral adipocyte-associated DEGs were validated in human hypertrophic cardiomyopathy using single-cell sequencing data. **(A)** The overall clustering of cells in human hypertrophic cardiomyopathy. **(B)** The hub genes were validated to be highly expressed in patients with human hypertrophic cardiomyopathy, including OGN, SELL, FOS, NKG7, LOX, HBB, CXCL9, CP and ALOX5.

**Figure 5:**
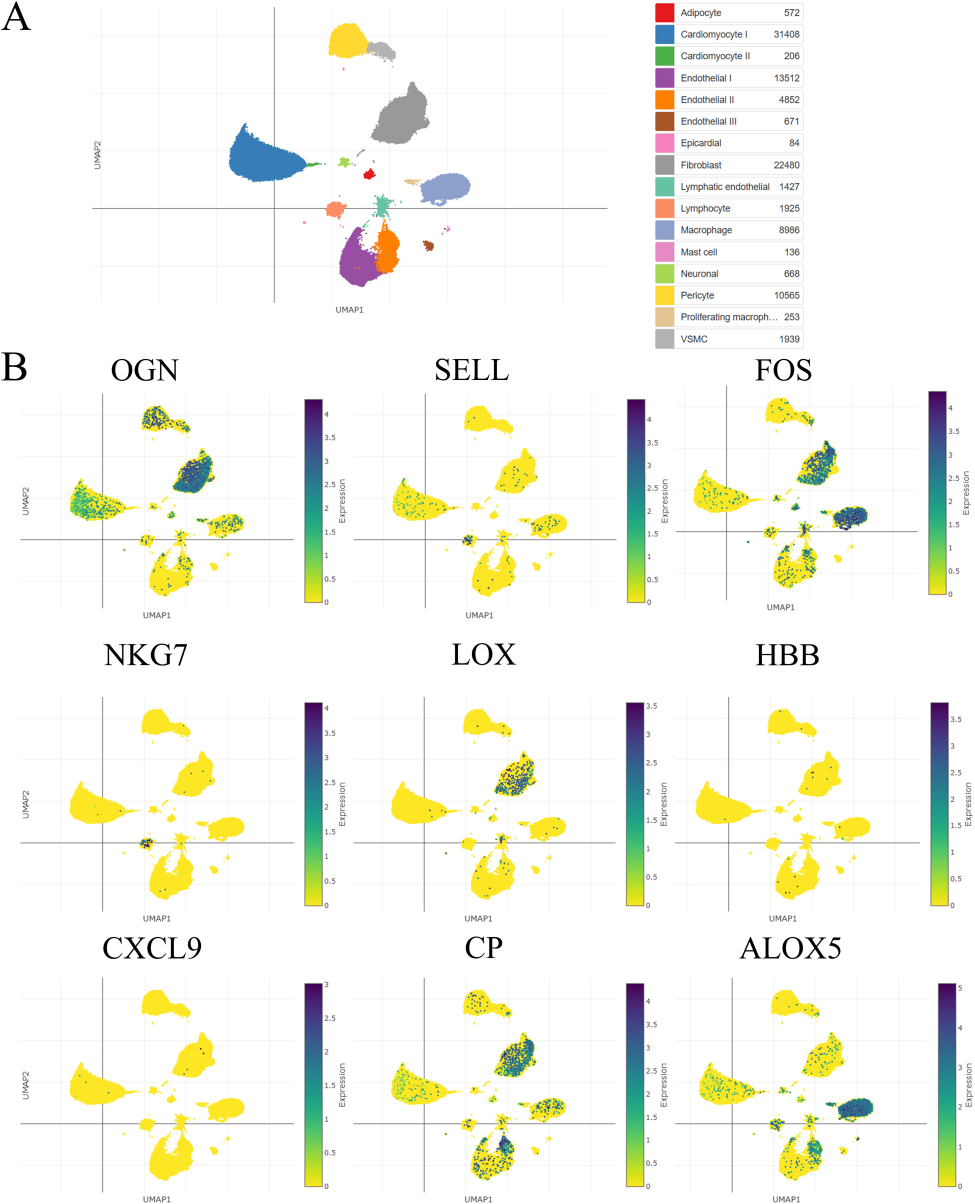
The hub visceral adipocyte-associated DEGs were validated in ischemic cardiomyopathy patients with end-stage heart failure using single-cell sequencing data. **(A)** The overall clustering of cells in ischemic cardiomyopathy patients with end-stage heart failure. **(B)** The hub genes were validated to be highly expressed in ischemic cardiomyopathy patients with end-stage heart failure, including OGN, SELL, FOS, NKG7, LOX, HBB, CXCL9, CP and ALOX5.

Using a single-cell portal, sequencing of hub visceral adipocyte-associated gene expression demonstrated that the hub genes were highly expressed in human hypertrophic cardiomyopathy and ischemic cardiomyopathy patients with end-stage heart failure, respectively. (Figures 4, 5), which may be potential targets for heart failure progression, especially HFpEF. As shown in Figures 4, 5, FOS and ALOX5 were mainly expressed in macrophages and OGN was mainly expressed in fibroblasts, which were all significantly expressed in disease progression and required more attention to explore the effects on heart failure.

### 3.4 Hub gene validation in human sub-epicardial tissues and human blood samples

**Figure 6:**
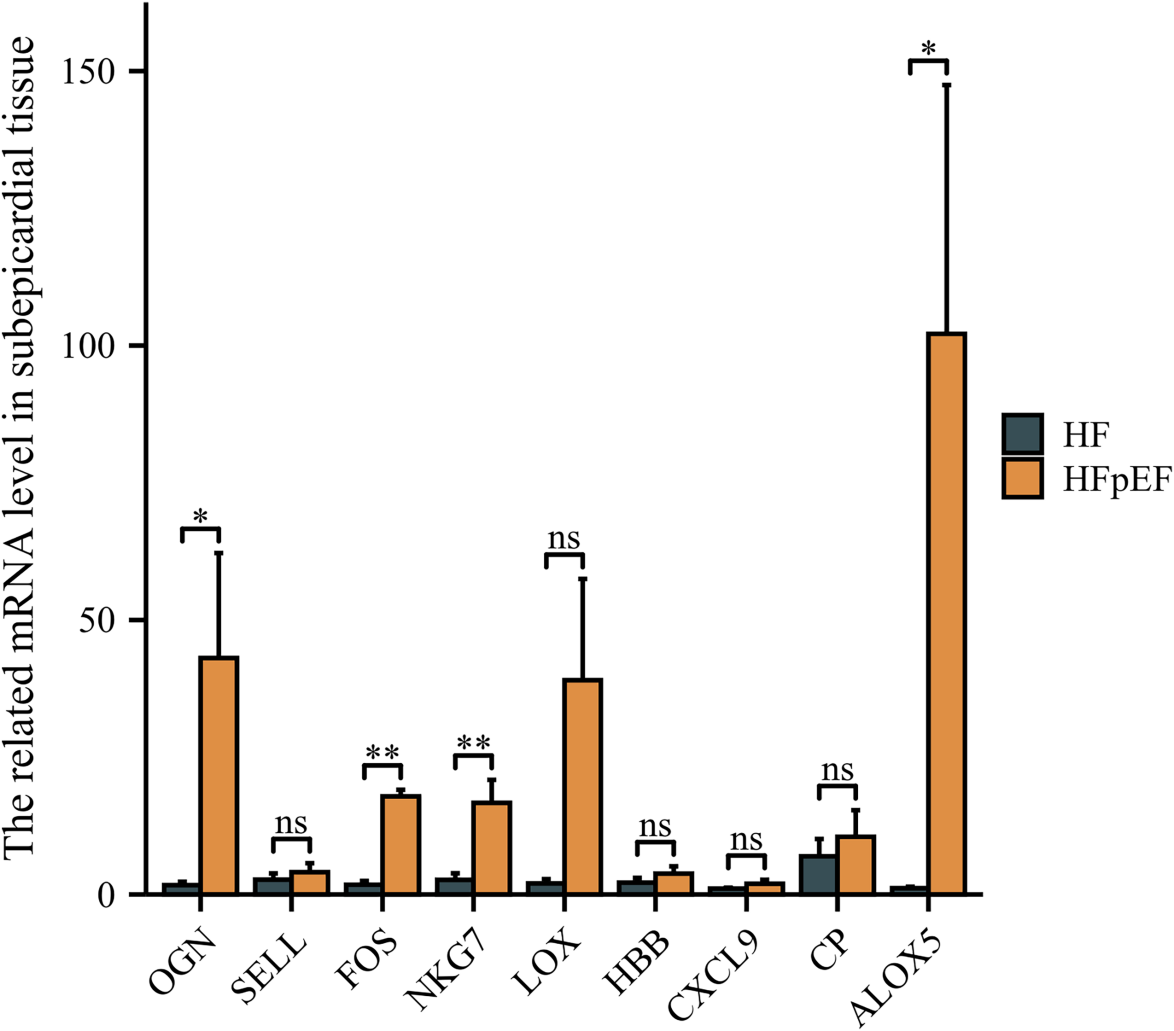
The validation of hub genes expression using human sub-epicardial tissues. qPCR was performed in human sub-epicardial tissues between HFpEF patients and heart failure patients. *n* = 10 per group. *P < 0.05; **P < 0.01; ns, not significant.

**Figure 7:**
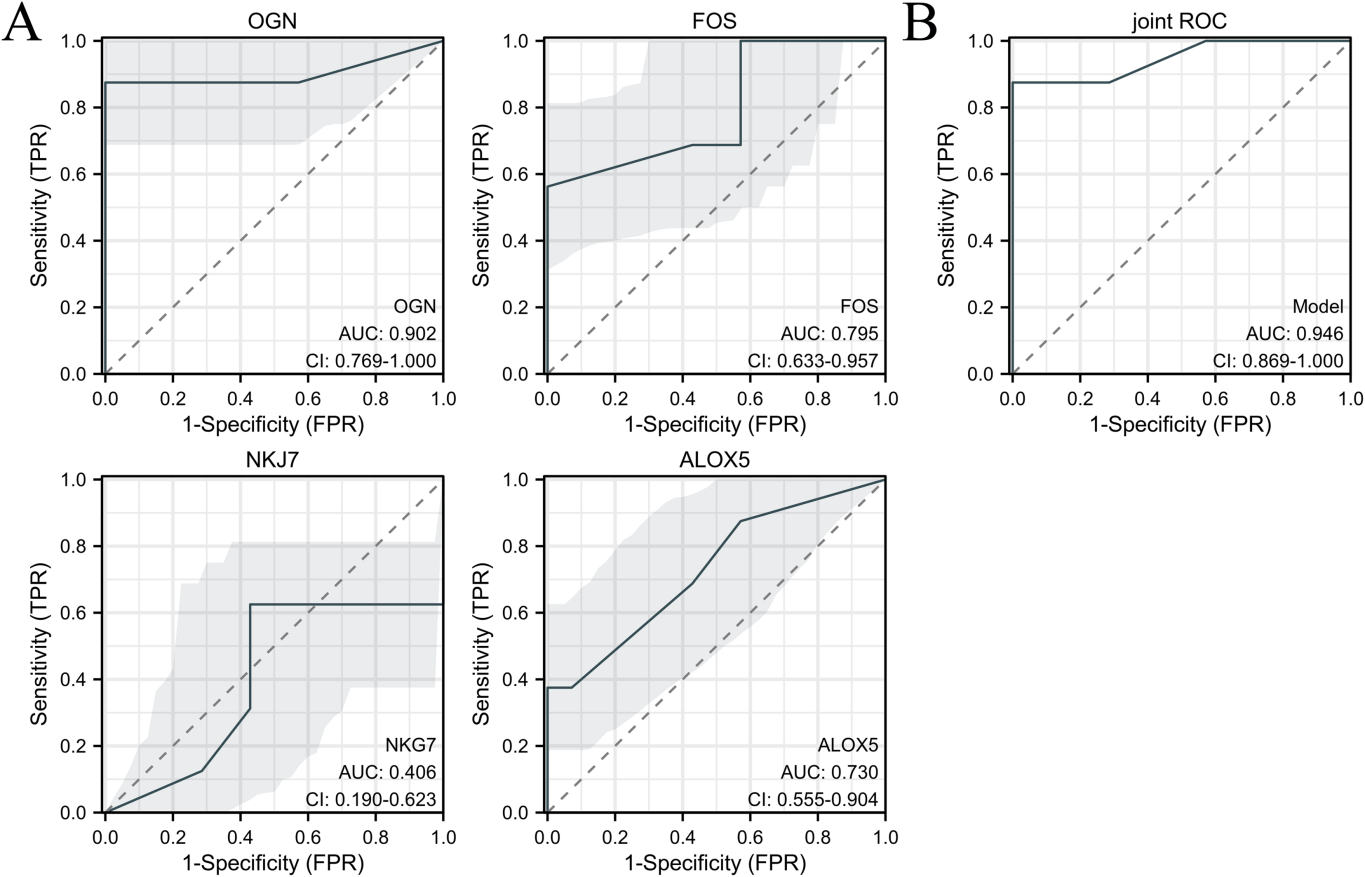
ROC analysis was performed in human blood samples to investigate the diagnostic value between HFpEF patients and heart failure patients. **(A)** ROC of OGN, FOS, NKG7 and ALOX5 expression between HFpEF patients and heart failure patients. **(B)** The joint ROC of OGN, FOS and ALOX5 expression between HFpEF patients and heart failure patients. *n* = 232 in HFpEF group and *n* = 32 in heart failure group.

The hub genes were validated using human sub-epicardial tissues who underwent HFpEF or OHF and need CABG surgery, demonstrating that the related OGN, FOS, NKG7 and ALOX5 mRNA expressions were highly expressed in sub-epicardial tissues in HFpEF patients compared to OHF patients (Figure 6). ROC was performed in human blood samples and the AUCs of OGN, FOS and ALOX5 were 0.902, 0.795 and 0.730, respectively (Figure 7). The AUC of joint ROC of OGN, FOS and ALOX5 was 0.946, suggesting that OGN, FOS and ALOX5 could be diagnostic markers between HFpEF patients and OHF patients.

There were no significant differences at baseline characteristics and laboratory examinations at 1 day after admission between HFpEF and OHF (Supplementary Table 4). The same were also with FOS, ALOX5 and OGN expression at 1 day after admission between HFpEF and OHF patients. We followed up the first admission HFpEF patients for 2 years recurrence, FOS, ALOX5 and OGN expressions were increased at follow up 1 year recurrence, while decreased at follow up 2 year recurrence (Supplementary Table 5). The Cox regression analysis of first AMI patients' follow up demonstrated that FOS, ALOX5 and OGN expressions could be risk factors for HFpEF disease progression (Supplementary Table 6).

### 3.5 Hub gene validation in cell lines

**Figure 8:**
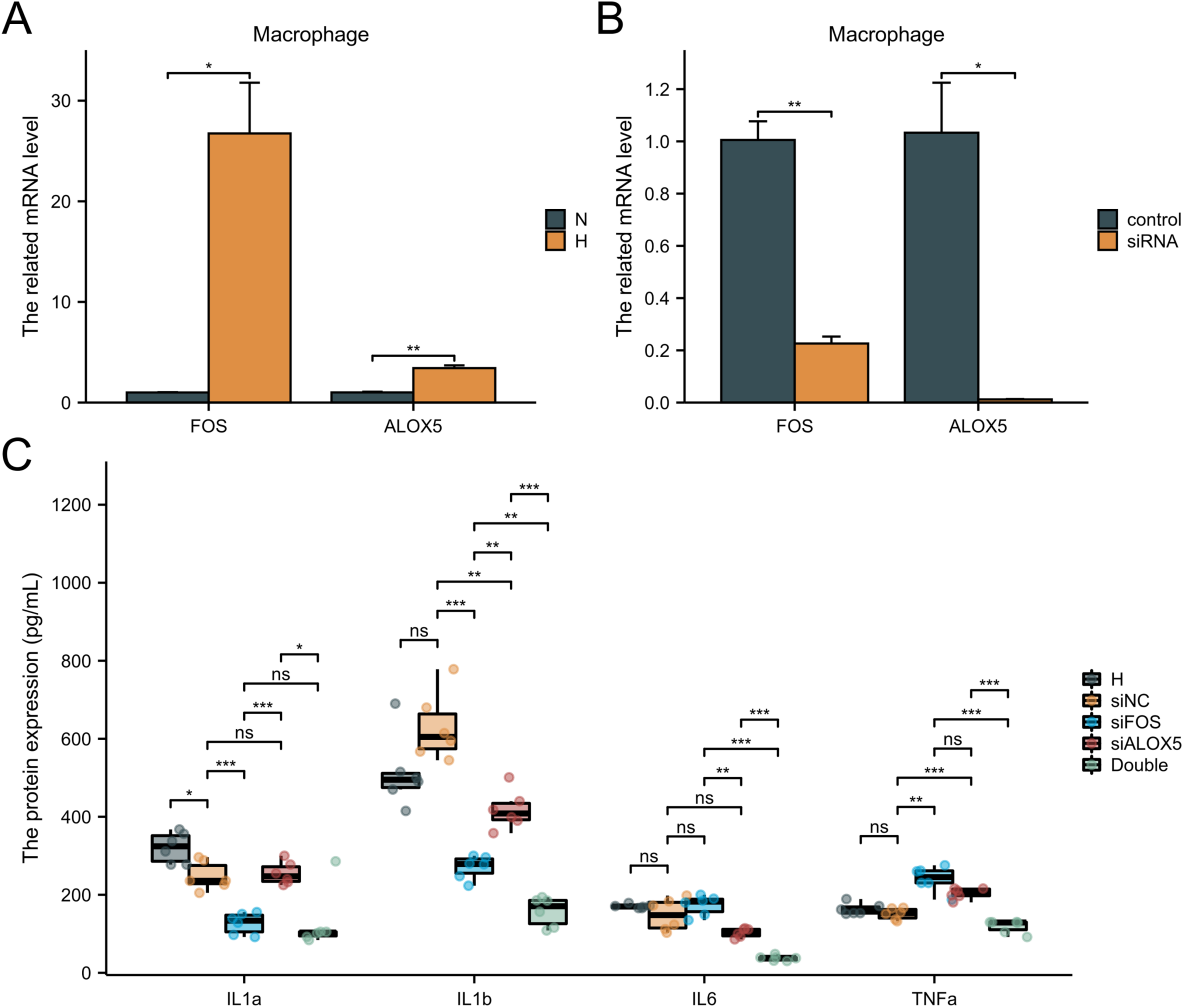
The effects of FOS and ALOX5 on macrophage senescence. **(A)** The FOS and ALOX5 related mRNA level in macrophage under hypoxia or normal. **(B)** The knockdown efficiency of FOS and ALOX5 siRNA. **(C)** ELISA was performed to determine the SASPs in macrophages under hypoxia and siRNA knockdown. *n* = 6 per group. *P < 0.05; **P < 0.01; ***P < 0.001; ns, not significant.

**Figure 9:**
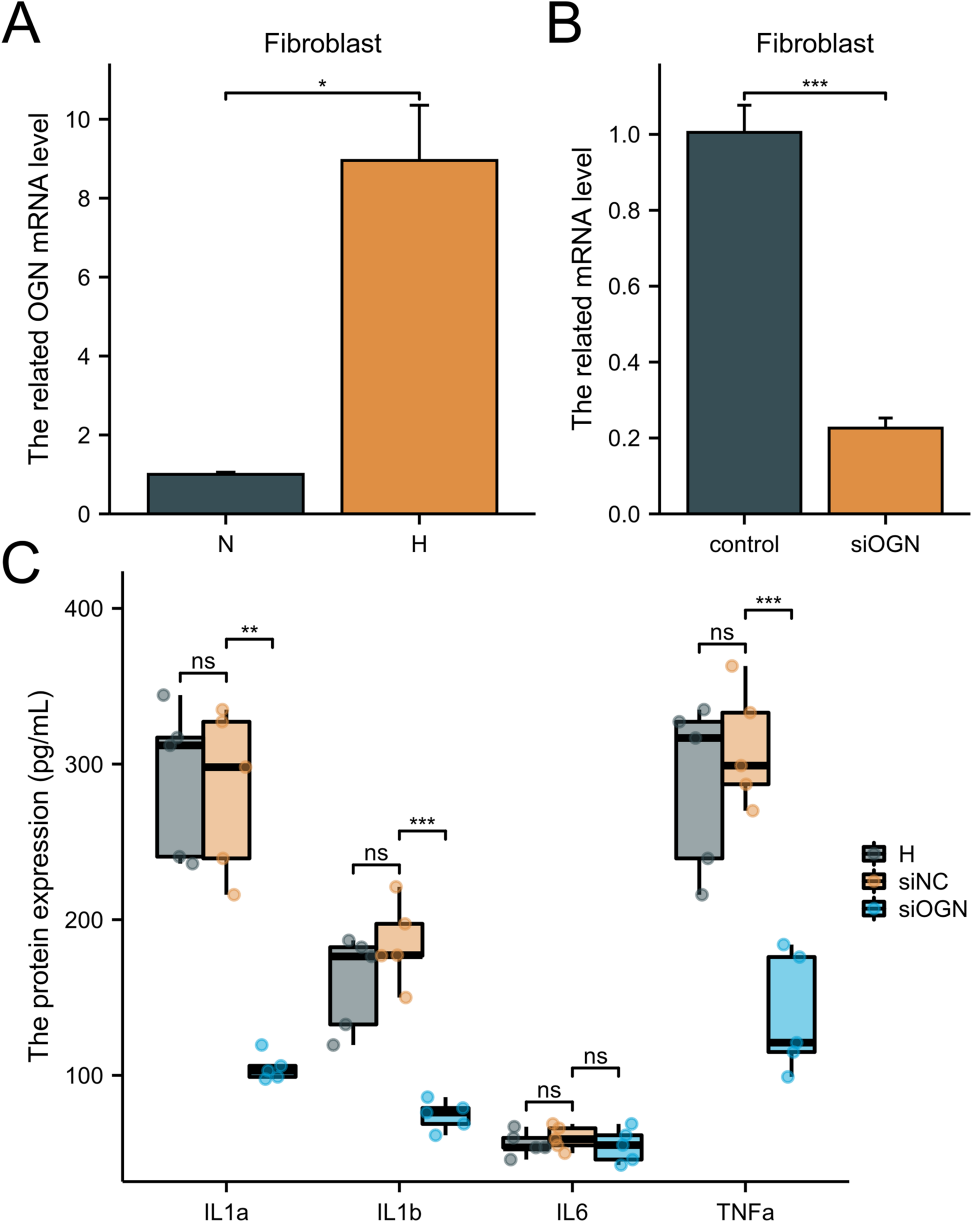
The effects of OGN on fibroblast senescence. **(A)** The OGN related mRNA level in fibroblast under hypoxia or normal. **(B)** The knockdown efficiency of OGN siRNA. **(C)** ELISA was performed to determine the SASPs in fibroblasts under hypoxia and siRNA knockdown. *n* = 6 per group. *P < 0.05; **P < 0.01; ***P < 0.001; ns, not significant.

To investigate the effects of the FOS, ALOX5 and OGN expression on HFpEF disease progression, macrophage and fibroblast cell lines were utilized. FOS and ALOX5 were highly expressed in macrophages under hypoxia, while OGN was highly expressed in fibroblasts under hypoxia (Figures 8A, 9A). SASPs, including IL1α, IL1β, IL6 and TNFα, decreased in hypoxic macrophages after FOS and ALOX5 knockdown or both (Figures 8B,C). Also, SASPs decreased in hypoxic fibroblasts after OGN knockdown (Figure 9B,C). These results suggested that FOS, ALOX5 and OGN may affect cell senescence after hypoxia, thus inducing myocardial infarction and HFpEF progression.

## 4 Discussion

The aetiology of heart failure is relatively clear, however, clinical treatment targets are still limited (19, 20). Subepicardial adipose tissue is correlated with obesity (BMI), left ventricular volume and fibrosis (21). Cellular senescence is generally an irreversible proliferative arrest in damaged normal cells that have exited the cell cycle (22). In 1961, Hayflick and Moorehead first studied the permanent arrest of proliferation at the cellular level. They found that the lifespan of primary human cells was limited to approximately 60 cell divisions (23). Cellular senescence can be divided into three main categories: (i) replicative senescence; (ii) developmentally programmed cellular senescence, evidenced to be a crucial process for healthy embryonic development; and (iii) stress-induced premature senescence, triggered by a wide range of external and internal by stimuli such as oxidative stress, oncogene expression, DNA damage (24).

Adipocytes and adipose-derived stem cells (ADSCs) impeded cardiac remodeling by regulating macrophage polarization through PI3K/STAT3 pathway after MI (25). Leptin promoted phosphorylation-STAT3 to bind to Ptgs2 promoter in cardiac myocytes (26) and macrophages (27). M2-like macrophage regulated Nrg1/ErbB signaling in fibrotic tissue formation after MI, which suppressed cardiac fibroblasts senescence (28). Macrophage migration inhibitor rejuvenated aged mesenchymal stem cells in human, thus improving myocardial repair after MI (29). In this study, visceral adipocyte-associated DEGs in heart failure were analyzed and the hub genes were obtained and validated using Single-cell sequencing data, human sub-epicardial adipose tissue and blood samples, and 3T3 and RAW 264.7 cell lines. We aimed to investigate the visceral adipocyte-associated hub genes, which contribute to cardiac fibrosis and inflamm-aging and result in heart failure deterioration.

Utilizing machine learning and network-based approach, OGN was identified as a biomarker covering multiple pathogenic pathways for diagnosing heart failure (30, 31). MI triggered prompt recruitment of neutrophils into murine hearts, which constituted the sequential cell-fate from naïve S100a4-positive, to activated Sell-high, to aging Icam1-high neutrophils (32). SELL expression was correlated to the mortality of patients with heart failure and T2 diabetes (33). c-fos and c-jun were markedly and immediately lowly expressed in the old rats than in the adult animals after heart failure (34). SIRT3 mediated the FOS inhibition through histone H3 deacetylation prevents cardiac fibrosis and inflamm-aging (35). The expression of LOX isoforms (LOX and LOXL1-4) was strongly increased upon MI, and this response was accompanied by a significant accumulation of mature collagen fibres in the infarcted area (36). LOX expression was observed in areas of extensive remodelling, partially overlapping with α-smooth muscle actin-expressing myofibroblasts. Tumour growth factor-β as well as hypoxia-activated pathways contributed to the induction of LOX expression in cardiac fibroblasts (37, 38). Multi-omics integration identified that HBB was highly associated with dilated, hypertrophic and ischemic cardiomyopathy as well as heart failure (39–41). CXCL9, as one of macrophage immune checkpoint proteins, controlled cardiac structure, signaling, and inflammation (42, 43). Alox5 belongs to a class of nonheme iron-containing dioxygenases involved in the catalysis of leukotriene biosynthesis, which was essential for biosynthesis of specialized pro-resolving mediators and cardiac repair in heart failure (44, 45). Cardiomyocyte-specific Alox5 depletion attenuated hypertensive ventricular remodeling. Conversely, cardiac-specifical Alox5 overexpression showed a pro-hypertrophic cardiac phenotype. Ablation of Alox5 in bone marrow-derived cells did not affect pathological cardiac remodeling and heart failure (44). The effects of NKG7 and CP expression on heart failure were less known, which required more attentions to illustrate the effects on cardiac remodeling and inflammation resolution.

There are some limitations. The sample size of human validation is quite small. The large, multi-center and double-blind cohort is still required to investigate the visceral adipocyte senescence effects on heart failure, especially HFpEF. The ability of these cytokines to prognosticate a large proportion of heart failure patients multivariately, encourages further studies to clarify the diagnostic and prognostic potential of cytokines in such patients.

## 5 Conclusion

Based on our current study, our research provided bioinformatics analysis of visceral adipocyte-associated DEGs biomarkers in the heart failure deterioration. The screened hub genes, including OGN, FOS and ALOX5, were validated using single-cell sequencing data, cell lines and human samples, which can be therapeutic targets for the treatment to cell senescence under hypoxia and prediction to heart failure progression to HFpEF.

## Data Availability

Publicly available datasets were analyzed in this study. This data can be found here: The data can be found here: https://www.ncbi.nlm.nih.gov/gds/?term=GSE251971.

## Supplementary material

Supplementary Figure 1 Identification of DEGs in chronic heart failure patients. **(A)** The volcano plot of the dataset demonstrated distinguished features between myocardial biopsies of patients with chronic heart failure induced by ischemic aetiology before and after treating with the OPT implantation. **(B)** The bar plot of myocardial biopsies of patients with chronic heart failure induced by ischemic aetiology before and after treating with the OPT implantation.

Supplementary Figure 2 The GO pathways enriched by DEGs in chronic heart failure patients. BP, biological process; CC, cellular component; MF, molecular function.
