# Supplementary figures and images for: Subepicardial adipose genes contribute to the deterioration of heart failure preserved ejection fraction
Source: Front Cardiovasc Med. 2025 Feb 21;12:1501397. doi: 10.3389/fcvm.2025.1501397 (PMC11885512; doi:10.3389/fcvm.2025.1501397)

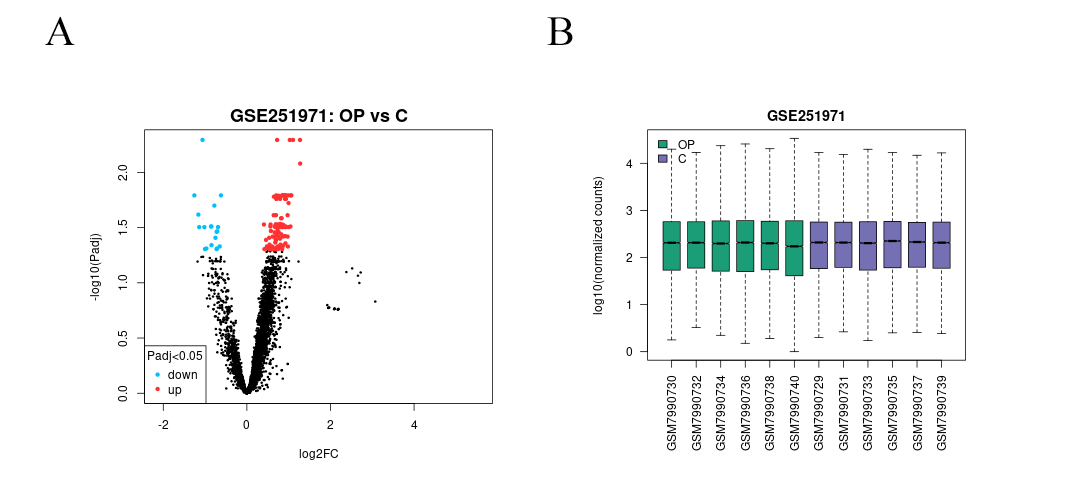

Supplement: Supplementary Figure 1 — Identification of DEGs in chronic heart failure patients. (A) The volcano plot of the dataset demonstrated distinguished features between myocardial biopsies of patients with chronic heart failure induced by ischemic aetiology before and after treating with the OPT implantation. (B) The bar plot of myocardial biopsies of patients with chronic heart failure induced by ischemic aetiology before and after treating with the OPT implantation. [file Image1.tif]

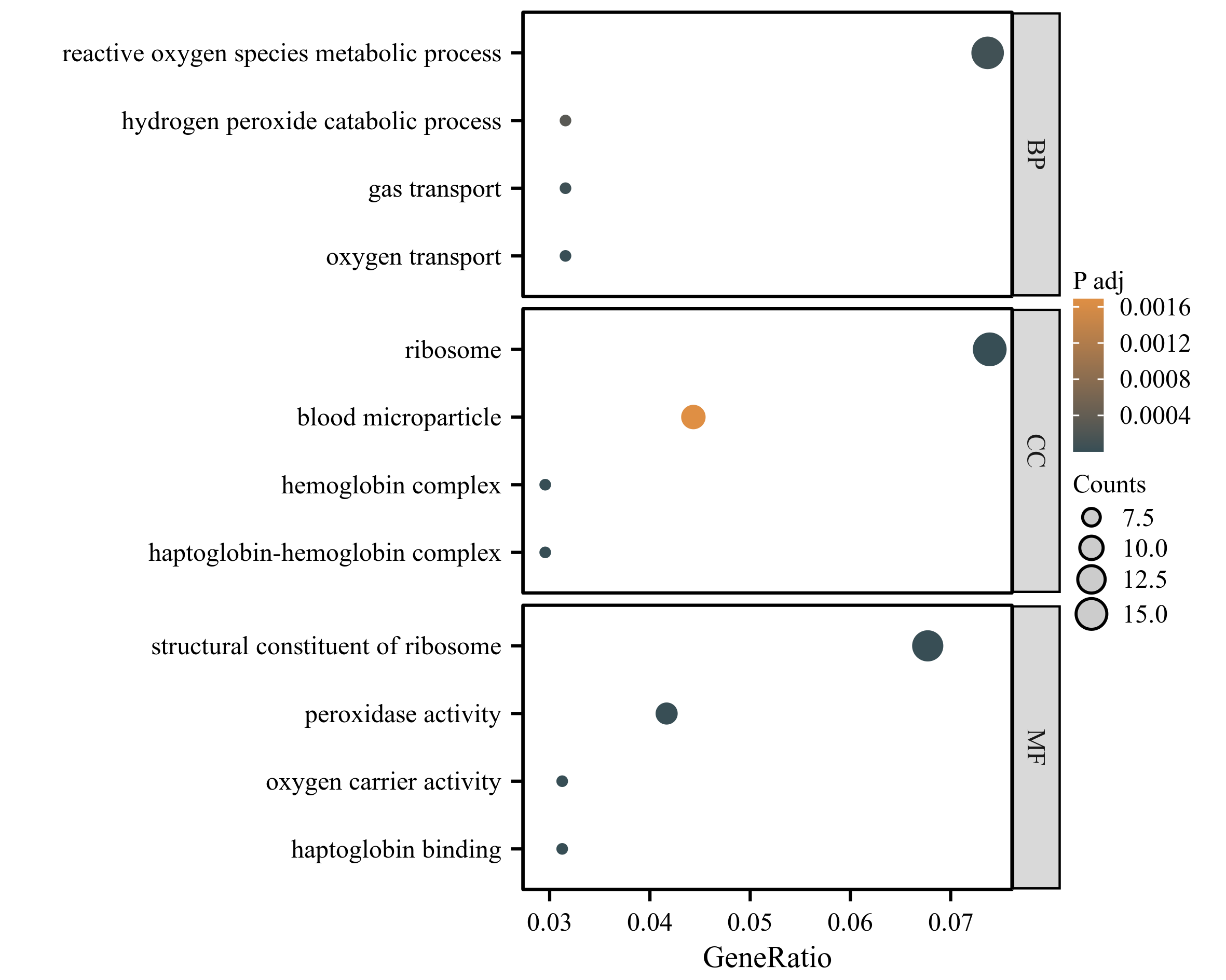

Supplement: Supplementary Figure 2 — The GO pathways enriched by DEGs in chronic heart failure patients. BP, biological process; CC, cellular component; MF, molecular function. [file Image2.tiff]
