# Supplementary material for: Subepicardial adipose genes contribute to the deterioration of heart failure preserved ejection fraction
Source: Front Cardiovasc Med. 2025 Feb 21;12:1501397. doi: 10.3389/fcvm.2025.1501397 (PMC11885512; doi:10.3389/fcvm.2025.1501397)
Supplement: Supplementary file 3 [file Datasheet1.pdf]

Supplementary Table 1. Specific human primers used for quantitative real-time PCR.

Genes	Forward (5’-3’)	Reverse (5’-3’)
OGN	TCTACACTTCTCCTGTTACTGCT	GAGGTAATGGTGTTATTGCCTCA
SELL	TGCCGAGACAATTACACAGATTT	TGAAAGGCAGAGTCTTCTCCAG
FOS	CACTCCAAGCGGAGACAGAC	AGGTCATCAGGGATCTTGCAG
NKG7	CATCATATCAGGCTACATCCACG	GCTGCGGTGGTTGAGACAA
LOX	GCCGACCAAGATATTCCTGGG	GCAGGTCATAGTGGCTAAACTC
HBB	AGGAGAAGTCTGCCGTTACTG	CCGAGCACTTTCTTGCCATGA
CXCL9	CCAGTAGTGAGAAAGGGTCGC	AGGGCTTGGGGCAAATTGTT
CP	GCCCTTTTTGGGCATATCTACT	CTGCCTGGACGTTGACGAA
ALOX5	ACTGGCTGAATGACGACTGG	CAGGGGAACTCGATGTAGTCC
β-actin	CATGTACGTTGCTATCCAGGC	CTCCTTAATGTCACGCACGAT

Supplementary Table 2. The significant GO and KEGG pathways enriched by heart failure DEGs.

Ontology	ID	Description	GeneRatio	BgRatio	pvalue	p.adjust
BP	GO:0015671	oxygen transport	6/190	15/18800	4.57e-09	1.13e-05
BP	GO:0015669	gas transport	6/190	21/18800	4.71e-08	5.84e-05
BP	GO:0072593	reactive oxygen species metabolic process	14/190	231/18800	1e-07	8.29e-05
BP	GO:0042744	hydrogen peroxide catabolic process	6/190	30/18800	4.78e-07	0.0003
BP	GO:0098869	cellular oxidant detoxification	9/190	100/18800	7.84e-07	0.0004
CC	GO:0031838	haptoglobin-hemoglobin complex	6/203	11/19594	5.08e-10	1.47e-07
CC	GO:0005833	hemoglobin complex	6/203	12/19594	1.01e-09	1.47e-07
CC	GO:0005840	ribosome	15/203	229/19594	1.73e-08	1.69e-06
CC	GO:0072562	blood microparticle	9/203	147/19594	2.31e-05	0.0017
CC	GO:0043020	NADPH oxidase complex	3/203	14/19594	0.0004	0.0214
MF	GO:0031720	haptoglobin binding	6/192	10/18410	2.41e-10	8.91e-08
MF	GO:0005344	oxygen carrier activity	6/192	14/18410	3.33e-09	6.15e-07
MF	GO:0003735	structural constituent of ribosome	13/192	181/18410	5.76e-08	5.67e-06
MF	GO:0004601	peroxidase activity	8/192	52/18410	6.15e-08	5.67e-06
MF	GO:0016684	oxidoreductase activity, acting on peroxide as acceptor	8/192	56/18410	1.12e-07	8.26e-06

GO, Gene Ontology; BP, Biological Process; CC, cellular component; MF, Molecular Function; KEGG, Kyoto Encyclopedia of Genes and Genomes.

Supplementary Table 3. The significant GO and KEGG pathways enriched by visceral adipocyte-associated DEGs.

Ontology	ID	Description	GeneRatio	BgRatio	pvalue	p.adjust
BP	GO:0006979	response to oxidative stress	10/64	433/18800	1.89e-06	0.0029
BP	GO:0000302	response to reactive oxygen species	7/64	203/18800	5.69e-06	0.0044
BP	GO:0072593	reactive oxygen species metabolic process	7/64	231/18800	1.32e-05	0.0059
BP	GO:0015671	oxygen transport	3/64	15/18800	1.66e-05	0.0059
BP	GO:0009636	response to toxic substance	7/64	247/18800	2.04e-05	0.0059
CC	GO:0031838	haptoglobin-hemoglobin complex	3/63	11/19594	5.13e-06	0.0005
CC	GO:0005833	hemoglobin complex	3/63	12/19594	6.83e-06	0.0005
CC	GO:0071682	endocytic vesicle lumen	3/63	23/19594	5.36e-05	0.0029
CC	GO:0072562	blood microparticle	5/63	147/19594	0.0001	0.0044
CC	GO:0062023	collagen-containing extracellular matrix	6/63	429/19594	0.0025	0.0807
MF	GO:0005506	iron ion binding	7/60	151/18410	5.83e-07	0.0001
MF	GO:0031720	haptoglobin binding	3/60	10/18410	3.89e-06	0.0004
MF	GO:0020037	heme binding	6/60	139/18410	5.95e-06	0.0004
MF	GO:0019825	oxygen binding	4/60	39/18410	7.7e-06	0.0004
MF	GO:0046906	tetrapyrrole binding	6/60	149/18410	8.87e-06	0.0004

Ontology	ID	Description	GeneRatio	BgRatio	pvalue	p.adjust
KEGG	hsa05143	African trypanosomiasis	4/39	37/8164	2.62e-05	0.0044

GO, Gene Ontology; BP, Biological Process; CC, cellular component; MF, Molecular Function; KEGG, Kyoto Encyclopedia of Genes and Genomes.

Supplementary Table 4. The characteristics of patients with OHF or HFpEF.

Characteristics	HFpEF	heart failure	P value
n	232	32	
gender			0.866
male	196 (84.5%)	24 75%)	
female	36 (15.5%)	8 (25%)	
age, year	60.7 ± 12.0	55.9 ± 12.0	0.294
BMI, kg/m2	25.3 (23.7, 28.2)	25.7 (23.5, 28.3)	0.746
smoke			1.000
No	148 (63.8%)	20 (62.5%)	
yes	84 (36.2%)	12 (37.5%)	
diabetes, year	1 (0, 6.5)	0.5 (0, 10)	0.951
encephalopathy			1.000
No	144 (62.1%)	20 (62.5%)	
yes	88 (37.9%)	12 (37.5%)	
CAD history			0.233
no	64 (27.6%)	0 (0%)	
yes	168 (72.4%)	32 (100%)	
PCI or CABG			0.308
no	52 (22.4%)	0 (0%)	
yes	180 (77.6%)	32 (100%)	
Systolic pressure, mmHg	130.2 ± 20.1	130.8 ± 21.8	0.940
diatolic pressure, mmHg	74.6 ± 8.5	77.9 ± 8.2	0.302
Left ventricular ejection fraction (%)	56.3 ± 2.4	35.1 ± 4.2	<0.0001
culprit vessel, n			0.707
LAD	104 (44.8%)	20 (62.5%)	
RCA	104 (44.8%)	8 (25%)	
LCX	12 (5.2%)	4 (12.5%)	
LCA	8 (3.4%)	0 (0%)	
D1	4 (1.7%)	0 (0%)	
treatment, n			0.582
PTCA	100 (43.1%)	16 (50%)	
PCI	104 (44.8%)	16 (50%)	
CABG	28 (12.1%)	0 (0%)	
TG, mmol/L	1.2 (1.0, 2.2)	1.6 (1.2, 2.0)	0.650
LDL, mmol/L	2.8 ± 0.9	3.2 ± 0.5	0.291
HDL, mmol/L	1.0 (0.9, 1.2)	1.0 (0.9, 1.1)	0.782
TC, mmol/L	5.1 ± 1.1	4.4 ± 1.4	0.181
LDH, mmol/L	418 (357.8, 785.8)	376 (296, 569.5)	0.283
HBDH, mmol/L	376.5 (337.3, 737)	357 (265.5, 533.5)	0.406
CK, ng/ml	706.5 (651.8, 1093.5)	717.5 (234.3, 1437.8)	0.783
CK-MB, ng/ml	76 (64.5, 112)	72 (30.5, 121)	0.523
cTNI, ng/ml	11.9 (8.8, 25.7)	11.4 (3.5, 30)	0.549
BNP, pg/ml	105 (76.6, 118.3)	102.5 (41.3, 167.2)	0.914

Characteristics	HFpEF	heart failure	P value
Uric Acid, mmol/L	292.5 (253.5, 337.5)	332 (260, 393.8)	0.160
serum creatinine, µmol/L	60 (55, 66.8)	73.5 (66, 83)	0.010
Fasting venous glucose, mmol/L	8.6 (7.5, 9.8)	7.065 (5.8, 9.6)	0.121
glycosylated hemoglobin, mmol/L	7.5 (6.8, 9.5)	6.9 (5.9, 9.7)	0.387
albumin, g/L	40.4 (36.8, 44)	39.4 (37, 42)	0.449
globulin, mg/L	27.1 ± 3.8	23.4 ± 4	0.017
TBIL, µmol/L	13.3 (10.1, 17.1)	12.25 (9.6, 16.7)	0.680
direct bilirubin, µmol/L	1.9 ± 0.7	1.7 ± 0.8	0.524
indirect bilirubin, µmol/L	11.8 (8.4, 15.1)	10.1 (8.4, 14.1)	0.702
ALP, U/L	85.5 (74.8, 107.5)	74 (60, 89.3)	0.191
ALT, U/L	56 (44, 61.8)	27.5 (19.3, 50.8)	0.026
AST, U/L	214.5 (145.3, 272.3)	107.5 (27.3, 173)	0.097
FOS, pg/mL	199.9 (184.7, 211.5)	199.6 (183.1, 214.9)	0.761
ALOX5, pg/mL	116.6 (101.1, 128.5)	113.65 (97.9, 130.7)	0.761
OGN, pg/mL	109.3 (89.3, 137.2)	109.08 (88.5, 139.9)	0.776

Supplementary Table 5. The characteristics of first AMI patients' follow up.

Characteristics	First AMI	1 year recurrence	2 year recurrence	P value
n	64	38	34	
Systolic pressure, mmHg	131.7 ± 18.3	117.6 ± 21.3	138.2 ± 22	0.009
diatolic pressure, mmHg	74.8 ± 7.4	71.3 ± 11.2	78 ± 7.7	0.074
single or not, n (%)				0.811
multivessel	56 (87.5%)	32 (84.2%)	30 (88.2%)	
single vessel	8 (12.5%)	6 (15.8%)	4 (11.8%)	
culprit vessel, n (%)				0.088
LAD	24 (37.5%)	26 (68.4%)	14 (41.2%)	
RCA	34 (53.1%)	10 (26.3%)	12 (35.3%)	
LCX	4 (6.3%)	2 (5.3%)	2 (5.9%)	
LCA	0 (0%)	0 (0%)	4 (11.8%)	
D1	0 (0%)	0 (0%)	2 (5.9%)	
treatment, n (%)				0.261
PTCA	26 (40.6%)	20 (52.6%)	12 (35.3%)	
PCI	28 (43.8%)	12 (31.6%)	22 (64.7%)	
CABG	8 (12.5%)	6 (15.8%)	0 (0%)	
TG, mmol/L	1.1 (0.9, 1.7)	1.3 (1.0, 1.6)	2.2 (1.4, 3.3)	0.006
LDL, mmol/L	2.8 ± 0.9	2.7 ± 0.9	3.1 ± 0.9	0.330
HDL, mmol/L	1 (0.9, 1.2)	1 (0.9, 1.19)	1 (0.8, 1.2)	0.946
TC, mmol/L	4.3 ± 1.2	4.5 ± 1.2	5 ± 1.7	0.301
LDH, mmol/L	376 (292, 538)	378 (294.5, 481)	500.5 (332, 655.5)	0.463
HBDH, mmol/L	357 (271, 414)	366 (271, 438.5)	462 (300.8, 630.5)	0.613
CK, ng/ml	685 (203, 1168.5)	756 (419, 924)	812 (659, 2248)	0.449
CK-MB, ng/ml	72 (22, 118)	71 (35, 83.5)	98 (61, 180)	0.466
cTNI, ng/ml	11.4 (4.9, 19.8)	9.3 (6.2, 26.3)	13.8 (9.4, 33.6)	0.482
BNP, pg/ml	102 (48.5, 157.6)	133 (45.2, 227)	68.3 (38.3, 115)	0.270
Uric Acid, mmol/L	336 (260, 393.5)	320 (268.5, 385.5)	310 (259, 362)	0.754
serum creatinine, µmol/L	74 (64.5, 86.5)	71 (66, 84)	69 (57, 80)	0.206
Fasting venous glucose, mmol/L	7.3 (6.2, 8.6)	7.6 (5.3, 9.9)	7.19 (6, 13.6)	0.787
glycosylated hemoglobin, mmol/L	6.7 (5.9, 9.8)	7.1 (6, 8.8)	7.4 (6.3, 9.9)	0.682
albumin, g/L	39.9 ± 3.6	37.2 ± 4.8	40.6 ± 3.9	0.025
globulin, mg/L	23 ± 4.2	24.5 ± 4.6	24.9 ± 3.9	0.279
TBIL, µmol/L	14.1 (10, 16.6)	10.8 (8.7, 13.5)	13.3 (10.8, 18.9)	0.215
direct bilirubin, µmol/L	1.7 ± 0.7	1.7 ± 0.8	2 ± 0.9	0.369
indirect bilirubin, µmol/L	11.5 (8.4, 14.4)	9.1 (8.1, 12)	9.9 (8.5, 15.3)	0.323
ALP, U/L	72 (61.5, 84.5)	73 (54.5, 89)	83 (71, 103)	0.070
ALT, U/L	27 (21.5, 52.5)	28 (16, 51)	38 (20, 60)	0.643
AST, U/L	126 (42.5, 184.5)	117 (56.5, 197)	101 (24, 235)	0.870
FOS, pg/mL	200.2 (190.8, 207.3)	272.37 (264.1, 277.6)	234.47 (215.6, 302.8)	< 0.001
ALOX5, pg/mL	116.4 (108.7, 120.3)	140.8 (134.3, 162.4)	86.6 (71.9, 97.1)	< 0.001
OGN, pg/mL	109.2 (101.5, 120.8)	154.1 (141, 186.7)	83.3 (79.6, 87.6)	< 0.001

Supplementary Table 6. The Cox regression analysis of first AMI patients' follow up.

Characteristics	Univariate analysis		Multivariate analysis	
	Odds Ratio (95% CI)	P value	Odds Ratio (95% CI)	P value
Systolic pressure	1.044 (1.011 - 1.077)	**0.008**	1.034 (0.997 - 1.072)	0.071
TG	1.380 (1.010 - 1.886)	**0.043**	1.402 (1.033 - 1.904)	**0.030**
albumin	1.200 (1.038 - 1.386)	**0.013**	1.188 (1.005 - 1.405)	**0.043**
ALP	1.027 (1.003 - 1.053)	**0.030**	1.022 (0.993 - 1.051)	0.137
FOS	2.034 (1.997 - 2.072)	**0.002**	1.044 (1.011 - 1.077)	**0.008**
ALOX5L	0.983 (0.967 - 1.000)	**0.050**	0.980 (0.926 - 1.038)	0.492
OGN	0.988 (0.975 - 1.001)	0.070	0.994 (0.949 - 1.040)	0.790
